# Low-spin ferric iron in primordial bridgmanite crystallized from a deep magma ocean

**DOI:** 10.1038/s41598-021-98991-w

**Published:** 2021-09-30

**Authors:** Yoshiyuki Okuda, Kenji Ohta, Yu Nishihara, Naohisa Hirao, Tatsuya Wakamatsu, Sho Suehiro, Saori I. Kawaguchi, Yasuo Ohishi

**Affiliations:** 1 Department of Earth and Planetary Sciences, Tokyo Institute of Technology, Tokyo, 152-8550 Japan; 2 Geodynamics Research Center, Ehime University, Ehime, 790-8577 Japan; 3 Japan Synchrotron Radiation Research Institute, Hyogo, 679-5198 Japan; 4 Present Address: Department of Earth and Planetary Sciences, Graduate School of Science, The University of Tokyo, Bunkyo, Tokyo 113-0033 Japan

**Keywords:** Solid Earth sciences, Geophysics, Mineralogy

## Abstract

The crystallization of the magma ocean resulted in the present layered structure of the Earth’s mantle. An open question is the electronic spin state of iron in bridgmanite (the most abundant mineral on Earth) crystallized from a deep magma ocean, which has been neglected in the crystallization history of the entire magma ocean. Here, we performed energy-domain synchrotron Mössbauer spectroscopy measurements on two bridgmanite samples synthesized at different pressures using the same starting material (Mg_0.78_Fe_0.13_Al_0.11_Si_0.94_O_3_). The obtained Mössbauer spectra showed no evidence of low-spin ferric iron (Fe^3+^) from the bridgmanite sample synthesized at relatively low pressure of 25 gigapascals, while that directly synthesized at a higher pressure of 80 gigapascals contained a relatively large amount. This difference ought to derive from the large kinetic barrier of Fe^3+^ rearranging from pseudo-dodecahedral to octahedral sites with the high-spin to low-spin transition in experiments. Our results indicate a certain amount of low-spin Fe^3+^ in the lower mantle bridgmanite crystallized from an ancient magma ocean. We therefore conclude that primordial bridgmanite with low-spin Fe^3+^ dominated the deeper part of an ancient lower mantle, which would contribute to lower mantle heterogeneity preservation and call for modification of the terrestrial mantle thermal evolution scenarios.

## Introduction

The early state of the Earth’s mantle was likely to be fully molten due to the radiogenic, core formation^1^, and the widely believed Moon-forming giant impact^2–4^. The terrestrial deep magma ocean evolved into the present form of the Earth’s mantle through core-mantle differentiation, mineral crystallization, and phase changes. High-pressure melting experiments showed that bridgmanite, an iron and aluminium (Fe,Al)-bearing MgSiO_3_ perovskite, is the first crystallizing phase for melts created by partial melting of pyrolitic mantle throughout lower mantle pressures^5,6^. Recent mantle dynamics simulations have shown that primordial bridgmanite-enriched domains^7^ can persist throughout Earth’s history if they have a distinct density and viscosity difference from the surroundings^8,9^. Heterogeneity preservation is meaningful for explaining the observed primordial isotopic fingerprints^10,11^, and its investigation requires the detailed physicochemical and rheological properties of bridgmanite.

**Figure 1:**
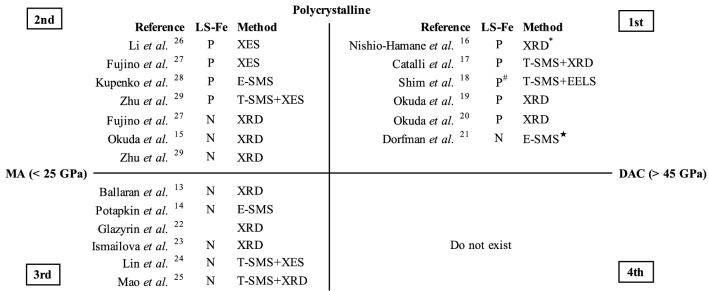
The spin state of Fe in (Fe,Al)-bearing bridgmanite compiled in a two-dimensional diagram with the x-axis the sample synthesis pressure and the y-axis the sample grain size. 1st and 2nd quadrants, studies used a multigrain sample, 3rd and 4th quadrants, studies used a single-crystal sample. 1st and 4th quadrants, sample synthesized in a DAC, 2nd and 3rd quadrants, used a sample pre-synthesized in a MA. Numbers in the table denote the quadrants. ^*^: discussed in Fujino et al.^20^. ^#^: lack clear evidence from their synchrotron Mössbauer spectroscopy (SMS) results but has suggested being present from their Electron Energy-Loss Spectroscopy (EELS) results. *: hiroseite with almandine composition. *P* present; *N* no evidence. T- and E-SMS, time- and energy-domain SMS, respectively.

The electronic spin state of iron in bridgmanite controls various physical and chemical properties of the lower mantle, which is the most voluminous and massive layer on Earth. Since the first experimental demonstration by Badro et al.^12^, numerous studies have investigated the mechanism of the spin transition of bridgmanite samples with various chemical compositions and its influence on the properties of bridgmanite^13–29^ (see Lin et al.^30^ for a review). However, whether iron and aluminium (Fe,Al)-bearing bridgmanite undergoes a spin transition in the lower mantle has remained elusive for decades. Those synthesized at relatively low pressure in a multi-anvil apparatus (MA) show no evidence of the spin transition^13–18^, implying a high-spin Fe throughout the lower mantle, which is consistent with theoretical studies^31–34^. On the other hand, other experiments showed the spin transition of ferric iron (Fe^3+^) in octahedral sites (Si sites) of bridgmanite directly transformed at high pressure in a diamond anvil cell (DAC)^19,24–28^ (Fig. 1).

One way to interpret this contradiction is by attributing the invisible spin transition^13–18^ to the difficulty of achieving equilibrium in the site distribution of Fe^3+^ due to the large kinetic barrier of cation exchange^20–22,25^ described below, which is suggested to be a plausible process for bridgmanite containing low spin (LS) Fe^3+^ to be generated from initial high spin (HS)^20^.

1 $$ {\text{Fe}}_{{{\text{A}},{\text{HS}}}}^{{3 + }}  + {\text{Al}}_{{\text{B}}}^{{{\text{3}} + }}  \Leftrightarrow {\text{Al}}_{{\text{A}}}^{{{\text{3}} + }}  + {\text{Fe}}_{{{\text{B}},{\text{LS}}}}^{{{\text{3}} + }} $$

However, direct spectroscopic observation of the Fe spin state of (Fe,Al)-bearing MgSiO_3_ bridgmanite directly synthesized in a diamond anvil cell (DAC) above 45 GPa is so far limited to 1 report^25^, making the spin state of Fe in bridgmanite unclear. Examining the aforementioned hypothesis is of great importance for determining the spin state of primordial bridgmanite crystallized from an ancient deep magma ocean and hence its thermal evolution because the proposed scenarios of magma ocean crystallization lack information on the spin state of bridgmanite and its effect^35–37^.

**Figure 2:**
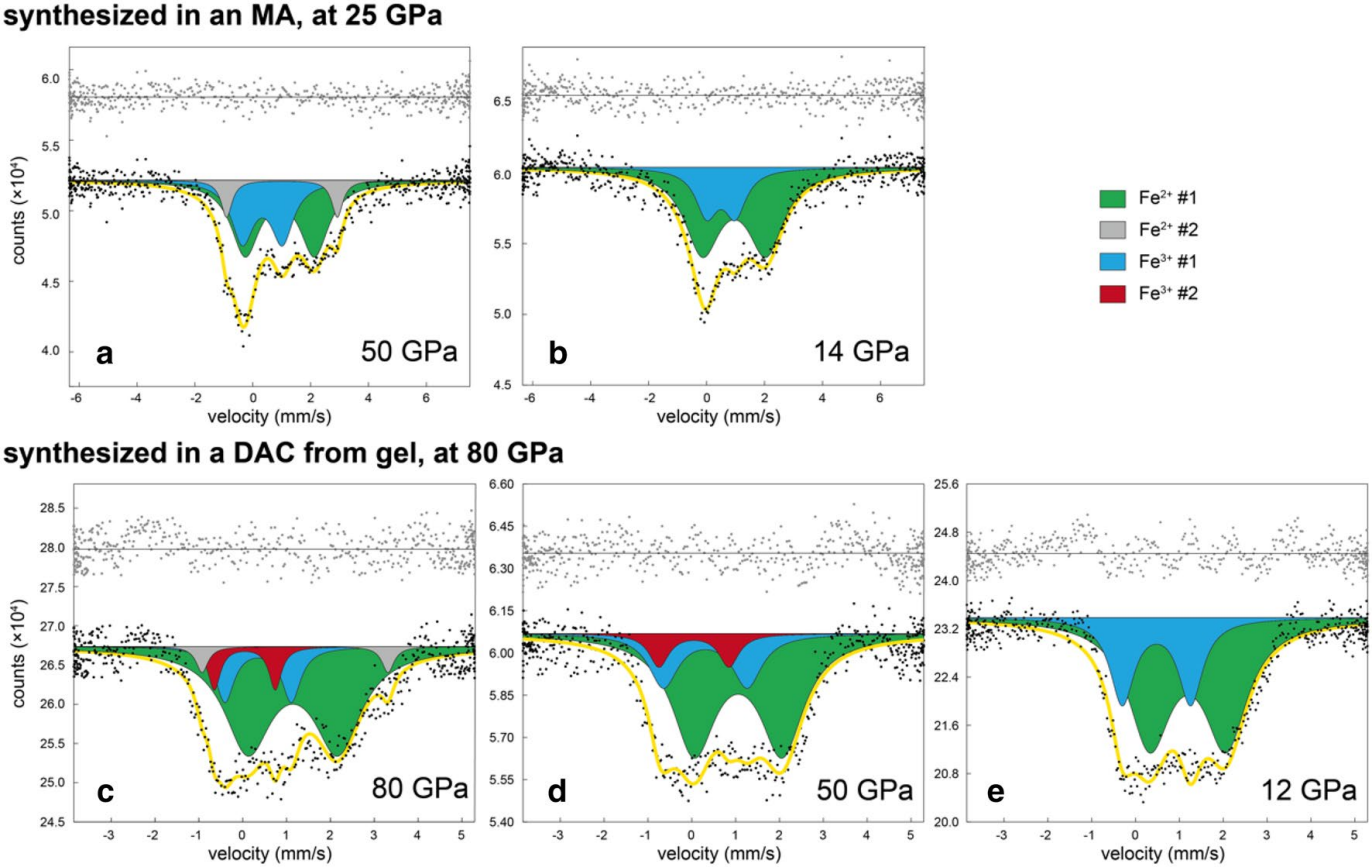
Evolution of the obtained SMS spectra of bridgmanite samples with decreasing pressure. (**a**,**b**) Bridgmanite synthesized in an MA at 25 GPa and 1673 K (MAOS3265) at 50 and 14 GPa, respectively, and (**c**–**e**) bridgmanite synthesized in a DAC at 80 GPa and 2500 K (DACMSRd01) at 80, 50, and 12 GPa, respectively. Green, grey, light blue, and red doublets indicate the components Fe^2+^ #1, Fe^2+^ #2, Fe^3+^ #1, and Fe^3+^ #2, respectively. Yellow curves show the theoretical fit, and grey symbols indicate the residual.

Here, we designed a comparative experiment on two different bridgmanite samples utilizing room-*T* energy-domain SMS combined with a DAC technique that is best suited for the analysis of iron distributions between individual crystal sites in bridgmanite and its Fe^3+^ content and electronic states at high pressures^14,21,38^. The bridgmanite samples were synthesized from the same gel starting material with a chemical composition of Mg_0.78(3)_Fe_0.13(1)_Al_0.11(1)_Si_0.94(2)_O_3.03(1)_. A sample, MAOS3265, was a single crystal bridgmanite synthesized in an MA at 25 GPa and 1673 K (Figs. S1b and S2c) and then loaded into DAC for a series of SMS experiments. Another sample, DACMSRd01, was a polycrystalline bridgmanite directly synthesized from the gel in a DAC at 80 GPa and 2500 K (Figs. S1a and S2a). Mössbauer spectra of MAOS3265 were obtained at pressures of 50 and 14 GPa, and those of DACMSRd01 were obtained at pressures of 80, 50, and 12 GPa (Fig. 2). Before the SMS experiments at 50 and 80 GPa, we performed laser thermal annealing at about2000 K and 2500 K, respectively, to release deviatoric stress in both samples (Table S1).

## Results

**Figure 3:**
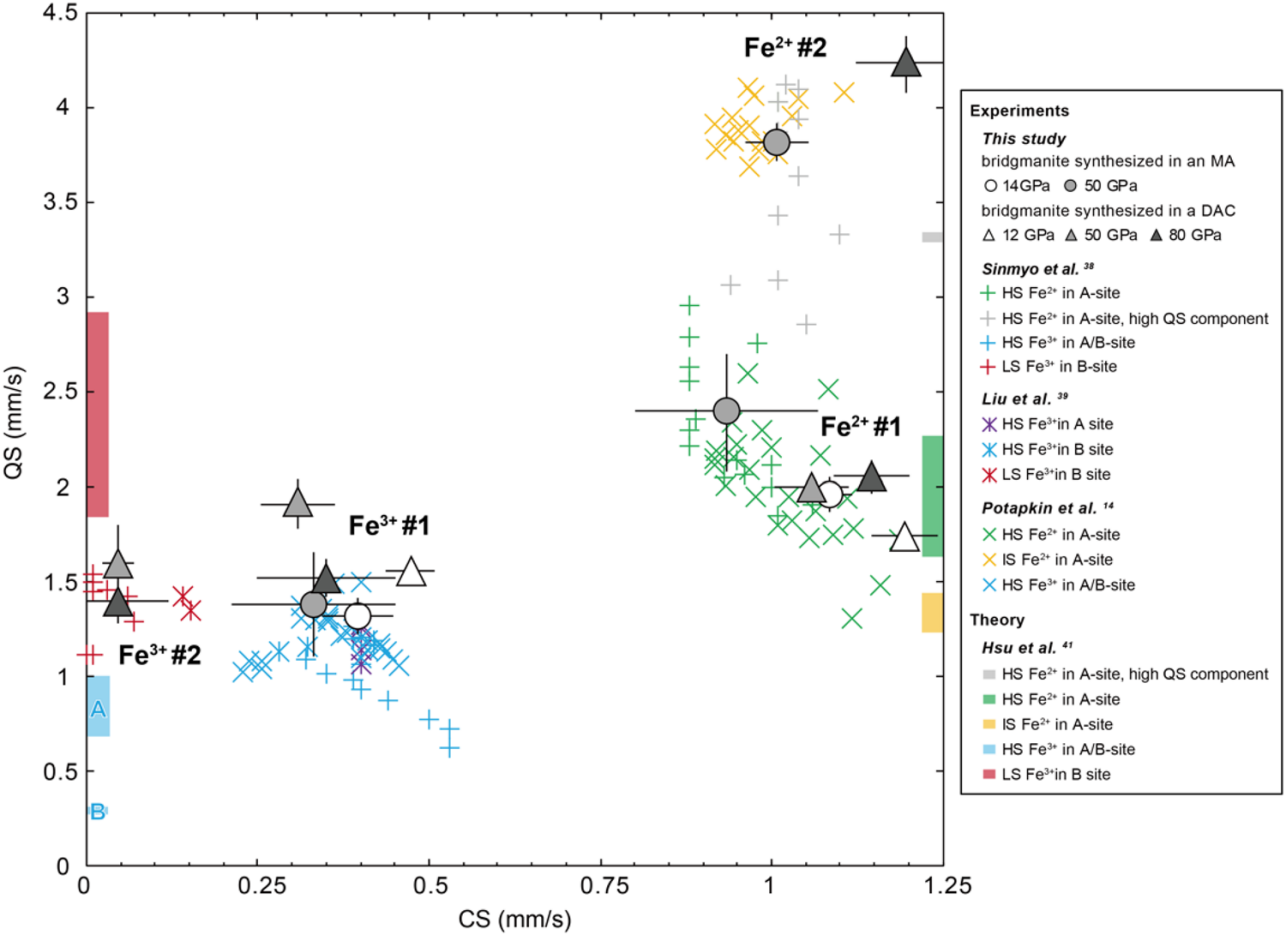
Mössbauer parameters of bridgmanite. Triangles, bridgmanite synthesized in a DAC (this study); circles, bridgmanite synthesized in an MA (this study). The reported Mössbauer parameters of bridgmanite are also plotted as a reference: plus symbols, Sinmyo et al.^38^; stars, Liu et al.^39^; crosses, Potapkin et al.^14^, respectively. The differences in colours represent the differences in assigned sites in previous studies: green, grey, yellow, light blue, and red symbols indicate the Mössbauer parameters assigned to HS Fe^2+^ in the A site, HS Fe^2+^ in the A site with a high QS component, intermediate-spin (IS) Fe^2+^ in the A site, HS Fe^3+^ in the A/B site, and LS Fe^3+^ in the B site, respectively. Density functional theory results for Fe^3+^ are shown on the left, and those for Fe^2+^ are shown on the right^41^.

The Mössbauer spectrum of MAOS3265 bridgmanite at 14 GPa was well fitted by two Lorentz-type doublets, corresponding to Fe^2+^ having higher center shift (CS) and quadrupole splitting (QS) values (Fe^2+^ #1) than Fe^3+^ (Fe^3+^ #1) (Fig. 2b). These components showed negligible differences with increasing pressure to 50 GPa, with CS and QS values of 0.94(13) and 2.39(32) (Fe^2+^ #1) and 0.33(12) and 1.38(28) mm/s (Fe^3+^ #1), respectively (Fig. 3 and Table S2). We assigned these values, which were consistent with the reported hyperfine parameters, to high-spin (HS) Fe^2+^ and Fe^3+^^14,21,38,39^ in the A-site. A component with a high QS value (Fe^2+^ #2) was observed at 50 GPa, which was attributed to the Fe^2+^ in the distorted A site^21,38,40^ (Fig. 2a). We found no evidence of low-spin (LS) Fe^3+^ in our annealed MAOS3265 bridgmanite at 50 GPa, which is suggested to have a similar QS value as that of HS-Fe^3+^ but with relatively low CS value^21,38,39^.

The spectrum of bridgmanite sample DACMSRd01 at 12 GPa was composed of two components that were similar to those of MAOS3265 bridgmanite at 14 GPa (Fig. 2b,e). However, spectra at the higher pressures 50 and 80 GPa required another component of Fe^3+^ #2 with a low CS (~ 0.05 mm/s) for satisfactory fitting, which is likely to be attributed to LS Fe^3+^ in the B site, as found in previous studies^21,38,39^ (Figs. 2c,d and 3). The relative area of the LS Fe^3+^ component slightly decreased from 19.2 to 16.4% with decompression from 80 to 50 GPa, where the calculated LS fractions of Fe^3+^ (LS Fe^3+^/ΣFe^3+^) were both ~ 40%. Note that we did not observe a drastic increase in QS along the spin crossover that was predicted in a theoretical study^34^, which was also the case in previous experimental studies^38,39^. The pressure dependence of the Fe^3+^/ΣFe ratio in each bridgmanite sample was nearly constant throughout the experiment (Fig. S4), which is an expected result since we avoided thermal annealing during decompression except at the highest-pressure data points of 50 and 80 GPa for MAOS3265 and DACMSRd01 bridgmanite, respectively.

## Discussion

The present results at 50 GPa showed that bridgmanite synthesized at 25 GPa in an MA did not undergo the Fe^3+^ HS-LS spin transition, while bridgmanite formed directly from the amorphous starting material at 80 GPa in a DAC experienced the transition. We view our results for MAOS3265 bridgmanite in the following manner: when a single-crystal sample is used, i.e., under chemical composition-fixed conditions, the cation exchange reaction Eq. (1) is required for Fe^3+^ to be incorporated into the B site, but the reaction’s large kinetic barrier^20,21,25,42^ hampers achieving the equilibrated site configuration and thus the accommodation of LS Fe^3+^ even with further pressurization and annealing; on the other hand, the chemical composition and cation configuration of the DACMSRd01 sample were equilibrated since a direct synthesis of bridgmanite in a DAC at high pressure where Fe^3+^ is stable in the LS state does not require the cation exchange in Eq. (1) to accommodate LS Fe^3+^, which is consistent with the large LS fraction of Fe in bridgmanite formed in only a DAC at high pressure^25^. Our results sufficiently explain the decade of conflicting studies of the reported spin state of Fe in bridgmanite^13–29^ (Figure S8). Still, it is noted that this is inconsistent with a consensus made by theoretical studies that FeAlO_3_ is always energetically preferable to AlFeO_3_^31–34^ (see Supplementary S6 for further discussion). The electrical conductivity and the thermal conductivity of (Fe, Al)-bearing bridgmanite-dominant rocks that accompanied the Fe spin transition are all synthesized at relatively high pressure (> 45 GPa) in a DAC^27,43^, while those without it were synthesized in a MA^23,44^. The contradictory studies of the electrical conductivity^43,44^ and the thermal conductivity^23,27^ of (Fe, Al)-bearing bridgmanite-dominant rocks can also be explained by our results.

An SMS experiment on (Fe,Al)-bearing glass at room *T* with a similar chemical composition used in the present study did not show a spin transition^45^, indicating the importance of the thermal process or crystallization of bridgmanite for hosting LS-Fe in the SiO_6_ octahedra. We should note that glass with a substantial amount of Fe and Al such as Fe_3_Al_2_Si_3_O_12_^46^ and MORB composition^47^ are reported to contain LS-Fe without thermal process, which is still under debate and may be due to difference in chemical composition. The amorphous starting material is a good proxy of melt^47–50^, and we can expect bridgmanite crystallized from melt to have the same inheritance of the Fe spin state as in our study.

The density relationship among the forming minerals and the residual melts and their thermophysical properties govern the lower mantle dynamics. Our findings add information on the spin state of bridgmanite to the proposed evolution scenarios. The density and viscosity contrast between primordial material and pyrolite is suggested to enhance the preservation of primordial material^9^, which is robustly predicted from geochemical evidence^10,11^. A density contrast of more than ~ 0.4% seems to be required to preserve primordial domains during the long-term evolution of the Earth^9^, including a large blob primordial domain such as the bridgmanite-enriched ancient mantle structures (BEAMS)^8^. A HS-to-LS transition of Fe^3+^ in bridgmanite can result in a stronger density contrast (Supplementary S5); the spin transition of 10 mol% Fe^3+^ in bridgmanite causes ~ 0.6(2)% densification^51^, which favours a mantle convection model that preserves primordial material until today. Although the effect of the spin transition of Fe in bridgmanite on its viscosity is unknown, the second major lower mantle mineral, ferropericlase, shows a viscosity maximum at the depth corresponding to the onset pressure of the HS-to-LS transition of Fe of 40 GPa^52^. The viscosity of bridgmanite may behave similarly to that of ferropericlase. However, temperature is thought to enhance the spin transition pressure and widens the pressure interval^53,54^, which should weaken the effect of Fe spin transition in bridgmanite on its elasticity and viscosity than expected. We should also be aware that the temperature conditions in our study (~ 2500 K) covers most of the present mantle geotherm, but that of the early Earth’s mantle is thought to be much higher (~ 3500 K) so that the observed LS fraction of Fe^3+^ in bridgmanite may be different from that in early mantle bridgmanite. Since higher temperature enhances the stability field of HS^53^, verification along with quantitative data from high *P–T* experiments is required for further discussion.

Our study suggests that bridgmanite crystallized from the deep magma ocean contains LS Fe^3+^, whose effect on mantle dynamics has never been considered. The evolution and dynamics of the deeper part of the Earth’s lower mantle may have been controlled by the “denser” Fe,Al-bearing bridgmanite for over billions of years from the freezing stage of the magma ocean to the present.

## Methods

### Preparation of bridgmanite samples

The gel starting material was synthesized at the Tokyo Institute of Technology. The gel starting material was heated at 727 ℃ in a H_2_-CO_2_ gas-mixing furnace in which oxygen fugacity (*f*O_2_) was controlled to be slightly above the iron-wüstite (IW) buffer (Supplementary S2). The chemical composition of the starting material was determined to be Mg_0.78(3)_Fe_0.13(1)_Al_0.11(1)_Si_0.94(2)_O_3.03(1)_ by electron probe microanalyzer (EPMA) JXA-8530F in Tokyo Institute of Technology. ^57^Fe-enriched Fe powder (^57^Fe/total Fe ~ 96%) was used in the synthesis of the gel for high-pressure Mössbauer spectroscopy measurements.

The MAOS3265 sample was synthesized using a 2000-ton Kawai-type MA apparatus (ORANGE-2000) at Ehime University. The gel starting material was heated at 1000 °C in a H_2_-CO_2_ gas-mixing furnace to synthesize enstatite in which *f*O_2_ was controlled to be slightly above the IW buffer. The phase was identified by a micro section XRD measurement using Rigaku RAPID II-V/DW to be single-phase clinoenstatite (Fig. S2b). This was packed into a Re foil capsule and then loaded into a MgO container. In the synthesis experiment using the MA apparatus, an (Mg,Co)O octahedral pressure medium with an edge length of 10 mm and 8 tungsten carbide anvils with a truncation edge length of 4 mm were employed. Heating was performed for 2 h at 25 GPa and 1673 K, respectively, using a LaCrO_3_ heater. The synthesis temperature was monitored with a W3Re-W25Re thermocouple. As a result, we obtained an almost single-phase bridgmanite that was confirmed by XRD measurement using a Rigaku RAPID II-V/DW (Fig. S2c). The chemical composition was determined to be Mg_0.88(1)_Fe_0.13(1)_Al_0.11(1)_Si_0.91(1)_O_2.98(1)_ by EPMA. A single crystal was polished to a disk (~ 30 × 30 × 15 µm^3^) and loaded into a sample chamber in a DAC with a pressure medium of KCl, whose culet size was 300 µm (Fig. S1b). The pressure was determined from a Raman spectrum of the diamond anvil^55^. We used a typical value of 10% for pressure errors.

The disk-shaped gel starting material (~ 25 × 25 × 4 µm^3^) had gold sputtered on both sides as a laser absorber and was loaded into the sample chamber with a pressure transmitting medium of Al_2_O_3_ (Fig. S1a). The chemical composition of the sample in our previous studies with the use of Al_2_O_3_ pressure medium has never altered from that of the starting material^27,28^. The chemical composition of our bridgmanite sample before and after the experiment was analysed by EPMA JXA-8530F in the Tokyo Institute of Technology. We found that the chemical composition did not change before and after the experiment (Supplementary S1). Double-sided laser heating was conducted at BL10XU, SPring-8 to synthesize bridgmanite sample DACMSRd01. The size of the laser spot was larger than the sample size, and the entire gel starting material was converted into bridgmanite. Simultaneously, in situ XRD measurements were conducted to identify the crystalline phase with a 30 keV incident X-ray (Fig. S2a). We used a pair of bevelled diamonds with a culet size of 120 µm.

### Synchrotron Mössbauer spectroscopy at BL10XU, SPring-8

In situ high-pressure synchrotron Mössbauer spectroscopy measurements were all performed at BL10XU, SPring-8^56^. The X-ray energy was set to 14.4125 keV using double monochromatic Si(111) crystals, and the energy around the nuclear resonance energy of ^57^Fe was tuned using a high-resolution monochromator (HRM) consisting of nested-type channel-cut Si(511) and Si(975) crystals. A ^57^FeBO_3_ single-crystal heated to the Néel temperature in a 160 G external magnetic field was used for the nuclear monochromator, in which Bragg reflection (333) produced ultrafine monochromatic X-rays with an energy bandwidth of ~ 15 neV (calculated from the instrumental function) and an absorber bandwidth of 95% ^57^Fe-enriched K_2_MgFe(CN)_6_ of 0.197 mm/s. The ^57^FeBO_3_ crystal was mounted on a velocity transducer and operated in sinusoidal velocity mode. A NaI scintillation detector was used to count the ^57^Fe Mössbauer radiation. The collection time of the Mössbauer spectra was 4.5–16 h for each signal (Table S1). All measurements were carried out upon decompression from the highest pressure in each run. The obtained Mössbauer absorption spectra were fitted using a full transmission integral fit with Lorentzian-squared line shape using the MossA software package^57^.

## Supplementary Information

Supplementary Information.
