# Supplementary material for: Low-spin ferric iron in primordial bridgmanite crystallized from a deep magma ocean
Source: Sci Rep. 2021 Sep 30;11:19471. doi: 10.1038/s41598-021-98991-w (PMC8484549; doi:10.1038/s41598-021-98991-w)
Supplement: Supplementary file 1 — Supplementary Information. [file 41598_2021_98991_MOESM1_ESM.docx]

**Supplementary information for**

**Low-spin ferric iron in primordial bridgmanite crystallized from a deep magma ocean**

Yoshiyuki Okuda^1^*^†^, Kenji Ohta^1^, Yu Nishihara^2^, Naohisa Hirao^3^, Tatsuya Wakamatsu^1^, Sho Suehiro^1^, Saori I Kawaguchi^3^, and Yasuo Ohishi^3^

- ^1^: Department of Earth and Planetary Sciences, Tokyo Institute of Technology, Tokyo 152-8550, Japan
- ^2^: Geodynamics Research Center, Ehime University, Ehime 790-8577, Japan
- ^3^: Japan Synchrotron Radiation Research Institute, Hyogo 679-5198, Japan
- ^†^: Present affiliation, Department of Earth and Planetary Sciences, Graduate School of Science, The University of Tokyo, Bunkyo, Tokyo 113-0033, Japan

**S1. Chemical composition of the sample**

The chemical composition of the sample in our previous studies with the use of Al_2_O_3_ pressure medium has never altered from that of the starting material (Okuda et al., 2019, EPSL; Okuda et al., 2020, PCM). We observe that this is owing to the presence of Au layers preventing direct contact of the sample and the Al_2_O_3_.

To clarify the potential chemical reactions in our sample and Al_2_O_3_, we have checked the chemical composition of our bridgmanite sample before and after the experiment by electron probe microanalyzer (EPMA) JXA-8530F in Tokyo Institute of Technology. We found that the chemical composition did not change before and after the experiment (Table S3).

**
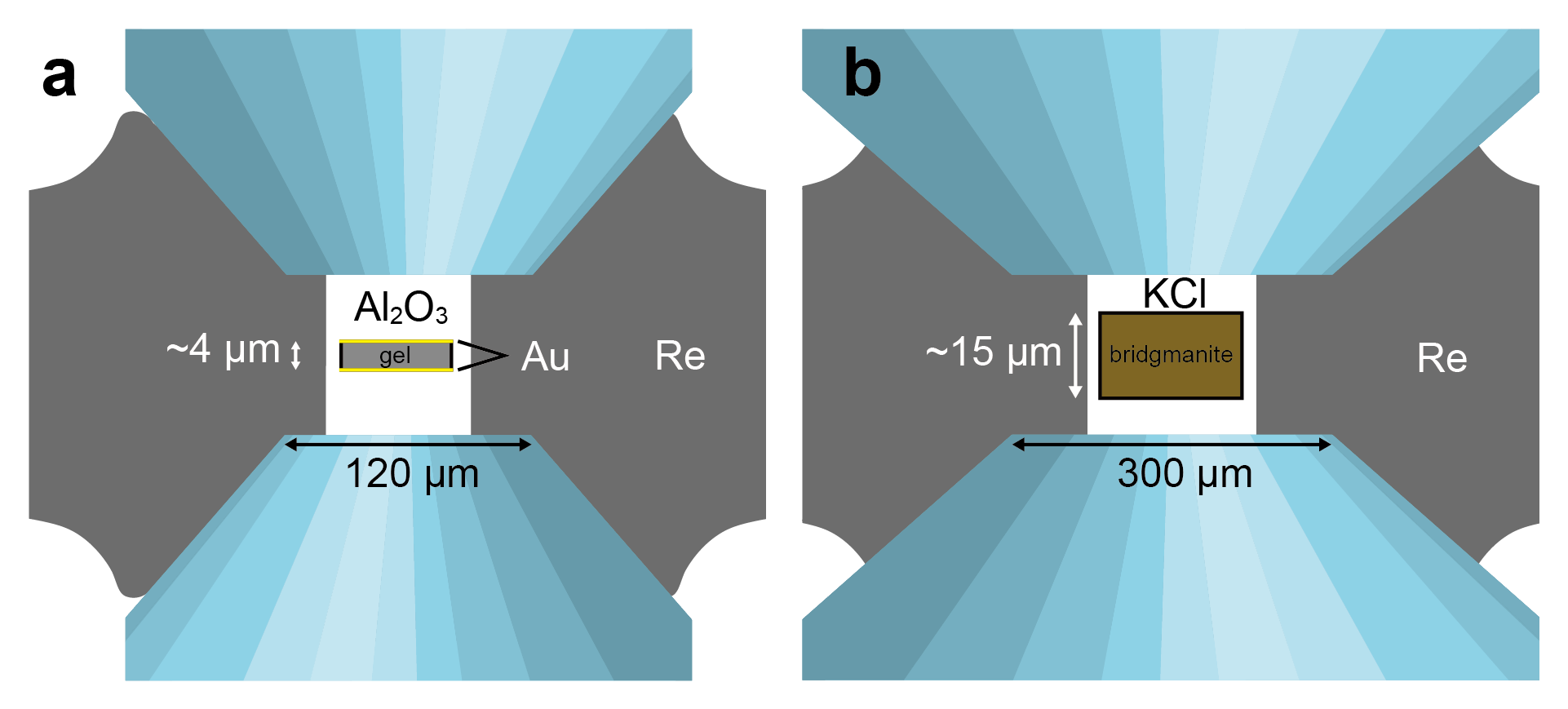
**

**Figure S1. DAC assembly of experiments with (a) DACMSRd01 and (b) MAOS3265.** The culets used for DACMSRd01 and MAOS3265 were 120 and 300 µm, respectively. To minimize the temperature gradient in the sample, we made the DACMSRd01 sample very thin, with an initial thickness of ~4 µm.

**
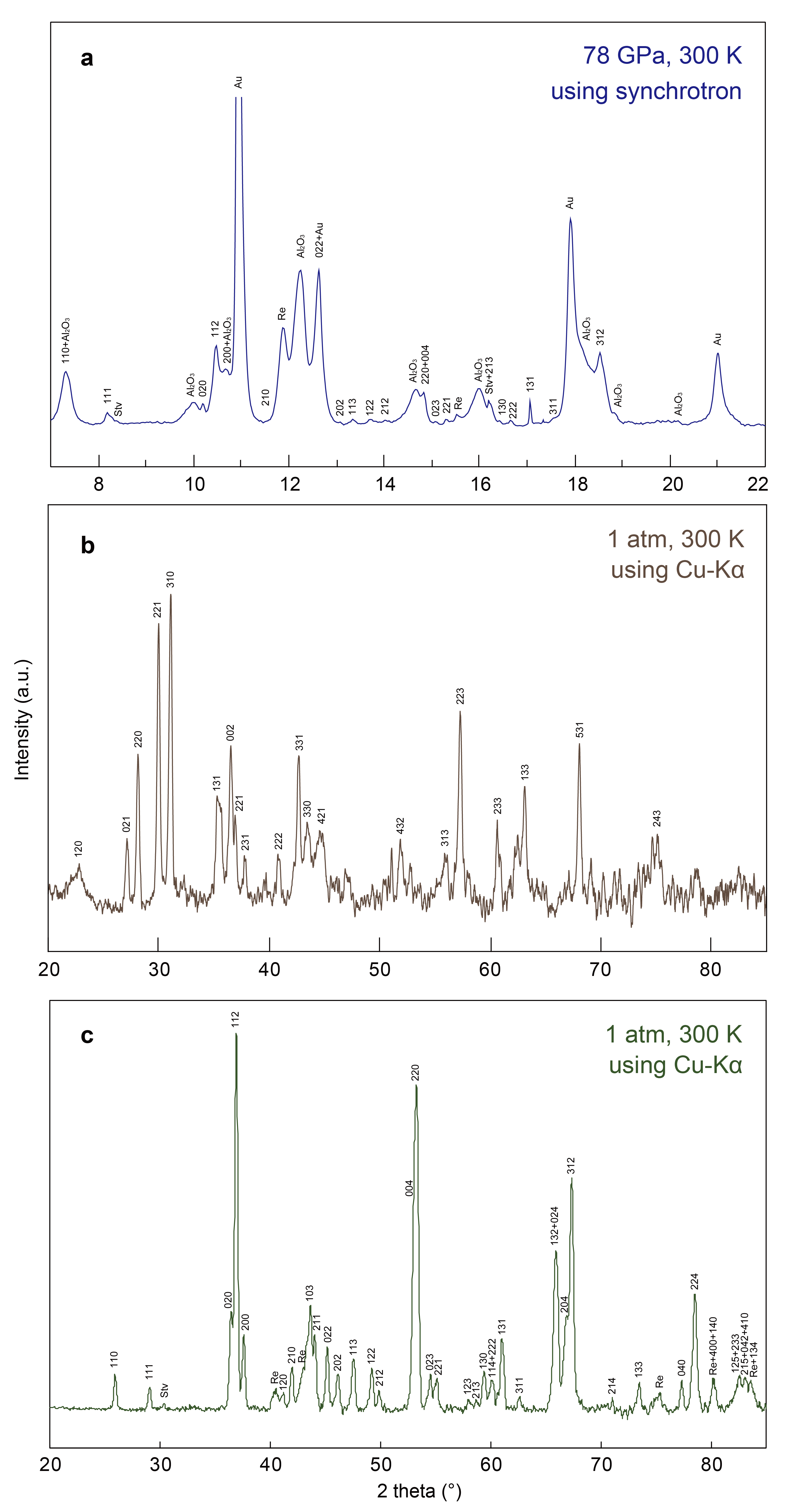
**

**Figure S2. XRD patterns of (a) bridgmanite sample DACMSRd01 immediately after its synthesis, (b) clinoenstatite as a starting material for MAOS3265 bridgmanite, and (c) recovered bridgmanite sample MAOS3265 pre-synthesized in an MA.** The Re peaks in Fig. S2a and Fig. S2b are from a Re capsule and from a Re gasket, respectively.


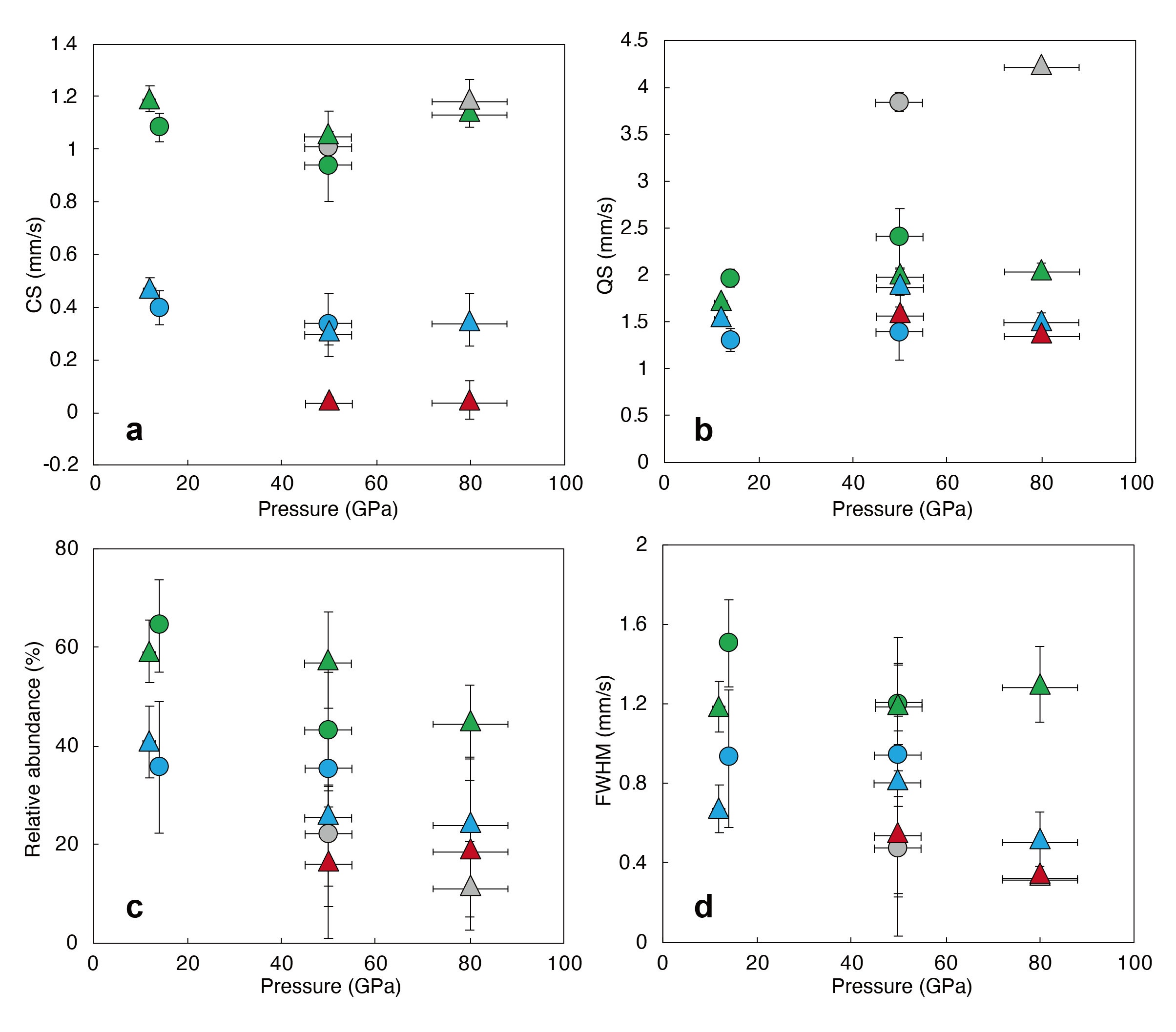


**Figure S3. Pressure dependence of Mössbauer parameters. (a) Centre shift, (b) quadrupole splitting, (c) relative abundance, and (d) FWHM.** Triangles, bridgmanite synthesized in a DAC (this study); circles, bridgmanite synthesized in an MA (this study). Green, grey, light blue and red symbols indicate components of Fe^2+^ #1, Fe^2+^ #2, Fe^3+^ #1, and Fe^3+^ #2, respectively.


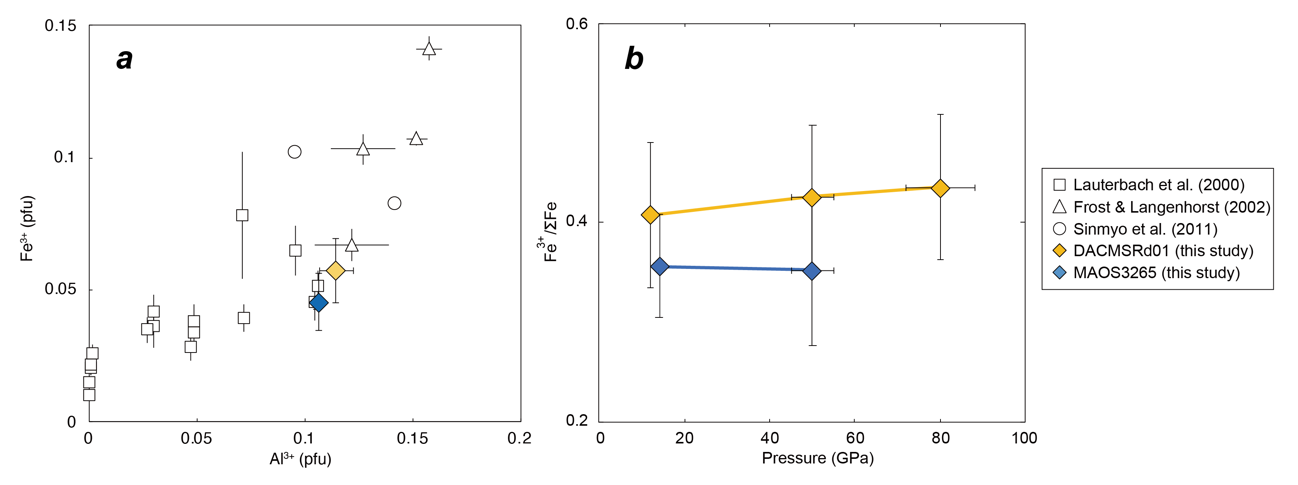


**Figure S4. (a) Fe^3+^ versus Al^3+^ content of bridgmanite, and (b) pressure dependence of the Fe^3+^/ΣFe ratio.** Yellow and blue diamonds, DACMSRd01 at 80 GPa and MAOS3265 at 50 GPa, respectively (this study); circles, Sinmyo *et al*. ^1^; squares, Lauterbach *et al*. ^2^; triangles, Frost and Langenhorst ^3^, respectively.

**S2. The valence state of Fe in the starting gel material**

We could not obtain the Mössbauer spectrum of the starting material due to the beamtime limitation. To minimize the temperature difference in the axial direction in laser heating, we chose to make the initial sample thickness less than 5 µm. However, reduced sample thickness requires a longer collection time of Mössbauer spectrum for high-quality data, which led to a relatively long collection time per data. The used gel starting material was reduced in an H_2_-CO_2_ furnace, controlling the *f*O_2_ slightly above the iron-wüstite buffer. The color of the powdered gel sample has changed from orange to grey, indicating a sufficient transition of ferric to ferrous iron (Fig. S5).


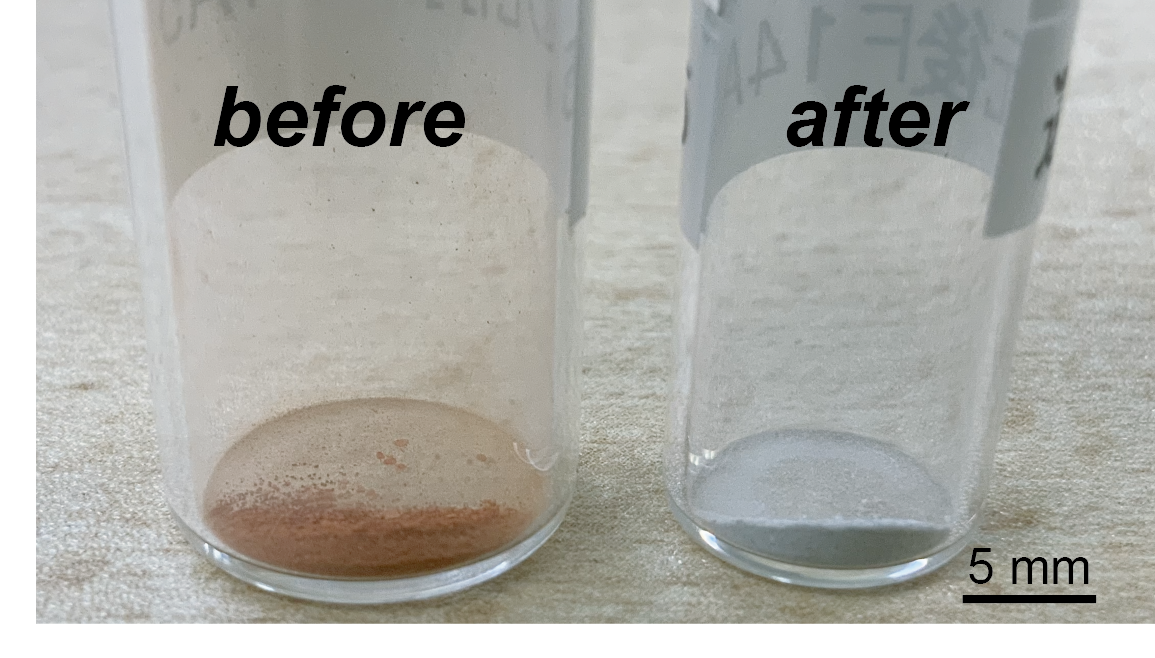


**Figure S5. Photograph of the gel starting material before (left), and after (right) the reducing procedure.**

**S3. Completeness of the transformation of a starting gel material to bridgmanite in a DAC**

The sample thickness was suppressed to ~5 µm for uniform heating of the axial direction, and the entire sample (~30×30 µm^2^) was heated using double-sided flat-top lasers with beam spot of > 30×30 µm^2^ for ~1 hr.

We used a similar experimental set up in our previous studies that measured thermal conductivity of bridgmanite (Okuda et al., 2019 EPSL) and post-perovskite (Okuda et al., 2020 EPSL). Since unreacted amorphous gel has extremely low lattice thermal conductivity, an unreacted gel sample should significantly affect the bulk thermal conductivity. We clarified that sample synthesis with sample thickness of thinner than 15 µm and heating duration of ~1hr is enough for a complete crystallization based on the collected XRD patterns, thermal conductivity values, and from finite element method (see Okuda et al., 2020 EPSL for details). Therefore, we deny an unreacted gel in our bridgmanite sample. **S4. Alternative fitting results**

In the main manuscript we have introduced three doublets to sufficiently fit our data. Here we provide an alternative fit on our data with two doublets (Fig. S6).

**Fig. S6. Mössbauer spectra fitted with two doublets.**

The yielded hyperfine parameters CS and QS of Fe^3+^ component from spectra at 50 and 80 GPa were 0.179 and 1.772, and 0.184 and 1.497, respectively. The CS values of the Fe^3+^ component for both 50 (pink plot) and 80 GPa (red plot) are much smaller than the suggested HS-Fe^3+^ value, indicating that there are not only HS-Fe^3+^ but also LS-Fe^3+^ in our sample (Fig. S7). Therefore, we are confident of the presence of LS-Fe^3+^ in our sample.


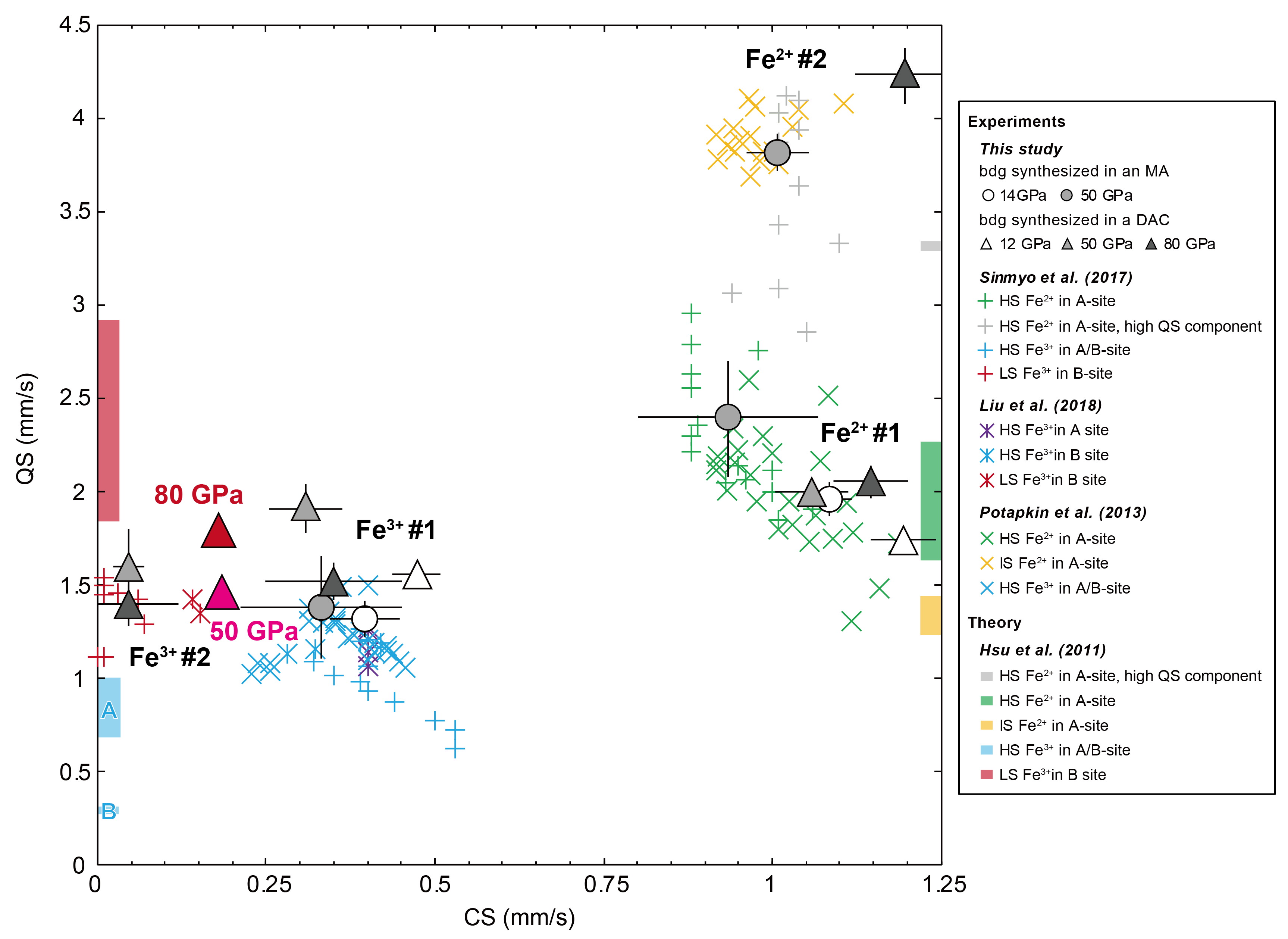


**Fig. S7. Hyperfine parameters obtained from two doublets fitting.**

**S5. The effect of the spin transition of Fe in bridgmanite on the density contrast created in the early mantle**

Whether primordial domains such as BEAMS (Ballmer et al., 2017) stabilize throughout the Earth’s history is suggested to be dependent on the density contrast between a shallow and deep region, rather than the actual density (Gülcher et al., 2019). Under a fractional crystallization, Caracas et al. (2019) showed that bridgmanite crystallizes at a shallower depth with increasing its Fe content as the magma ocean cools. This weakens the density contrast between a shallow and deep region. Although the amount of the density contrast in the mantle made by spin crossover is not huge as that that can be created by Fe content difference, since batch crystallization does not form a strong density difference and fractional crystallization even has a negative effect (Caracas et al., 2019), we expect that spin transition of Fe in bridgmanite influence creating density contrast in the lower mantle crystallized from the early magma ocean.

**S6. Supplementary Discussion**

It has been shown that an Al-free and Fe^3+^-bearing bridgmanite contains Fe^3+^ in the B site and undergoes a spin transition at high pressure ^4–12^. To our knowledge, Catalli *et al*. ^13^ first confirmed that (Fe,Al)-bearing bridgmanite can also contain LS Fe^3+^ in the B site by synchrotron Mössbauer spectroscopy (SMS). A subsequent experimental study ^14^ suggested that the LS Fe^3+^ content increases with pressure at high temperatures by cation exchange:

Fe^3+^_A,HS_+Al^3+^_B_ ⇔ Al^3+^_A_+Fe^3+^_B,LS_. (1)

However, further experimental studies denied the occurrence of such cation exchange ^15–17^, which was also supported by theoretical studies ^18–21^. Thanks to further studies, it became evident that a compressed single-crystal sample pre-synthesized in a multi-anvil press shows no evidence of LS Fe^3+^ ^15–17,22–24^, as the case of our sample MAOS3265. Conversely, polycrystalline bridgmanite samples show LS Fe^3+^, though interestingly, this result is probe-dependent; SMS and X-ray emission spectroscopy (XES) measurements detected this species ^14,25,26^, but XRD did not ^14,26,27^, indicating that such bridgmanite can accommodate only a small amount of LS Fe^3+^. However, bridgmanite synthesized in a DAC directly from an amorphous starting material at relatively high pressure was reported to contain LS Fe^3+^ in the B site, which was confirmed by both SMS and XRD ^13,28–31^. A two-dimensional compilation of results from previous studies and the present study regarding the spin state of (Fe,Al)-bearing bridgmanite is shown below (Figure S8). Note that Shim et al. ^31^ synthesized their bridgmanite sample in a DAC and found that the oxidation state of Fe in bridgmanite changes with changing *P-T* conditions. This was attributed to the spin transition of Fe^3+^, but they lacked direct evidence from their obtained Mössbauer spectra.

**Figure S8. The spin state of Fe in (Fe,Al)-bearing bridgmanite compiled in a two-dimensional diagram with the x-axis the sample synthesis pressure and the y-axis the sample grain size.** 1st and 2nd quadrants, studies used a multigrain sample, 3rd and 4th quadrants, studies used a single-crystal sample. 1st and 4th quadrants, sample synthesized in a DAC, 2nd and 3rd quadrants, used a sample pre-synthesized in a MA. Numbers in the table denote the quadrants. ^*^: discussed in Fujino et al. (2012). ^#^: lack clear evidence from their SMS results, but has suggested being present from their Electron Energy-Loss Spectroscopy (EELS) results. ^★^: hiroseite with almandine composition. P: present; N: No evidence. T- and E-SMS, time- and energy-domain SMS, respectively.

To interpret the previous reports, the chemical composition is one of the important factors because principally (Fe,Al)-bearing bridgmanite with more Fe^3+^ than Al must incorporate Fe^3+^ in the B site to maintain its charge balance. The Fe^3+^ content of the bridgmanite sample used in Catalli *et al*. ^13^ exceeded its Al content; thus, the observed LS Fe^3+^ in their sample might have been Fe^3+^ in the B site meta-stably trapped to preserve the charge balance. This is also true for Kupenko *et al*. ^25^, which had significantly higher Fe contents than other studies; note that the other previous studies had comparable Fe^3+^ and Al^3+^ contents ^13,14,26,29^ or Fe^3+^<Al compositions ^28,30^, so it is difficult to solely explain the conflicting results by chemical composition. Here, our gel starting material was rather enriched with Fe^2+^, and bridgmanite had had more Al than Fe^3+^. The results in our DACMSRd01 bridgmanite clarified that the B site in bridgmanite can accommodate LS Fe^3+^, even at Al/Fe^3+^>1. The most recent SMS study showed that Fe^2+^_0.55_Fe^3+^_0.12_Al_0.54_Si_0.73_O_3_ hiroseite, the Fe-analogue of bridgmanite, do not show spin transition ^32^. This may be due to the relatively larger A-site compared to bridgmanite that all the larger Fe atoms may have preferred A over B-site. Not only the Al/Fe^3+^ ratio but also the Fe content may largely affect the site occupancies in bridgmanite.

In addition to the chemical composition, the conflicting results in the 2nd and 3rd quadrants in Figure S8 were conventionally explained by thermal disorder. This stands in the point of view that Al^3+^ in the B site is sufficiently energetically preferable to both HS and LS Fe^3+^ there ^18–21^. The limited amount of LS Fe^3+^ located in the 2nd quadrant in Figure S8 that was confirmed by SMS measurements, e.g., ~5% ^25^, may be explained by the disorder caused by laser heating. The XES measurement performed by Fujino *et al*. ^14^ shown in the 2nd quadrant showed a higher relative abundance of ~20%, but we need to be aware that the collapse of the kβ’ peak in XES is also argued to be affected by its pressure-induced broadening under non-hydrostatic conditions ^17,24^. However, the different results located in the 1st and 3rd quadrants are unlikely to be explained by only the thermal disorder because of the relatively large LS fraction of Fe^3+^ shown in the studies in the 1st quadrant (Catalli *et al*. ^13^; this study). Likewise, all the reports located in the 1st quadrant involved gel or glass-state amorphous starting material so one may argue the unknown effect of the use of amorphous (i.e., having high entropy) material on the spin state and the formation of a metastable LS Fe^3+^ in B sites ^19^, but we do not believe that this is the case due to the observed large amount of LS Fe^3+^ in spite of the presence of the surrounding metallic Fe (also described later). Investigation of the spin state of (Fe,Al)-bearing bridgmanite directly synthesized not from gel or glass starting material but from oxide mixtures or mineral phases such as pyroxene or garnet in a DAC may clarify the effect of the use of amorphous starting material.

Zhu *et al*. ^26^ proposed that the kinetic barrier of cation exchange Eq. (1) can be large, which can also explain the undetected LS Fe^3+^ in studies located in the 3rd quadrant. This indicates the difficulty in achieving an equilibrium in the site distribution of Fe^3+^ even with thermal annealing, which was also suggested in a previous experimental study ^14^ and theoretical studies ^19,20^. This large kinetic barrier hypothesis can sufficiently explain the high LS Fe fraction in bridgmanite located in the 1st quadrant since that bridgmanite was directly synthesized from gel or glass-state amorphous starting material and hence did not require cation exchange Eq. (1) for accommodating LS Fe^3+^. In other words, the bridgmanite sample synthesized in an MA requires the cation exchange reaction for accommodating LS Fe in the B site since Fe^3+^ is initially HS and located in the A site, though Al^3+^ and Si are not thought to be energetically stable in the B site ^19,20^. As shown in Figure S8, a single crystal sample may have a larger kinetic barrier than a polycrystalline sample, which may be merely due to the crystal size difference, or reactions with other minor surrounding phases such as stishovite in the polycrystalline sample.

Here, the present experimental results strongly confirmed that bridgmanite directly synthesized at relatively high pressure can contain a certain amount of LS Fe^3+^; our DACMSRd01 bridgmanite synthesized from a reduced amorphous starting material well represents bridgmanite crystallized from a reduced magma ocean. The lower mantle bridgmanite crystallized from the magma ocean is also thought to coexist with metallic iron disproportionated from ferrous iron, which can be due to Fe^3+^-Al^3+^ ^33^ or Fe^3+^-Fe^3+^ ^34^ coupled substitution. Since our bridgmanite sample was synthesized from a well-reduced starting material, all the Fe^3+^ should have originated from the disproportionation of Fe^2+^ with the formation of metallic iron. When assuming Fe^3+^-Al^3+^ coupled substitution in our sample, since the Al^3+^ in the A site is thought to be unstable, the LS Fe^3+^ in the B site should react with the surrounding metallic Fe and transform to A-sited Fe^2+^. The fact that our DACMSRd01 bridgmanite crystallized from a Fe^2+^-enriched gel starting material contained a large amount of LS Fe^3+^ strongly indicates stable LS Fe^3+^ accommodation into bridgmanite, and supports the suggested disproportionation of ferrous iron in bridgmanite without the contribution of Al^3+^ ^34^.

To summarize, our study implies a disproportionation of Fe^2+^ to Fe^3+^ and metallic Fe in the ancient magma ocean with the contribution of the Fe^3+^-Fe^3+^ coupled substitution in bridgmanite ^34^, which crystallizes LS Fe^3+^-bearing bridgmanite and has profound implications for the Earth’s thermochemical evolution.

The present SMS measurement was conducted at only room temperature. Determining the spin transition depth in the actual lower mantle currently depends on theoretical studies ^35^ due to the lack of direct high-*P,T* spectroscopy measurements. Future studies on *in situ* SMS measurements of (Fe,Al)-bearing bridgmanite at high *P,T* can provide experimentally determined Fe spin transition depths in the lower mantle.

**Supplementary references**

1. Sinmyo, R., Hirose, K., Muto, S., Ohishi, Y. & Yasuhara, A. The valence state and partitioning of iron in the Earth’s lowermost mantle. *J. Geophys. Res. Solid Earth* **116**, 1–9 (2011).

2. Lauterbach, S., McCammon, C. A., van Aken, P., Langenhorst, F. & Seifert, F. Mössbauer and ELNES spectroscopy of (Mg,Fe)(Si,Al)O3 perovskite: a highly oxidised component of the lower mantle. *Contrib. to Mineral. Petrol.* **138**, 17–26 (2000).

3. Frost, D. J. & Langenhorst, F. The effect of Al 2 O 3 on Fe–Mg partitioning between magnesiowüstite and magnesium silicate perovskite. *Earth Planet. Sci. Lett.* **199**, 227–241 (2002).

4. Badro, J. *et al.* Electronic transitions in perovskite: Possible nonconvecting layers in the lower mantle. *Science.* **305**, 383–386 (2004).

5. Jackson, J. M. *et al.* A synchrotron Mössbauer spectroscopy study of (Mg,Fe)SiO 3 perovskite up to 120 GPa. *Am. Mineral.* **90**, 199–205 (2005).

6. Stackhouse, S., Brodholt, J. P. & Price, G. D. Electronic spin transitions in iron-bearing MgSiO3 perovskite. *Earth Planet. Sci. Lett.* **253**, 282–290 (2007).

7. Catalli, K. *et al.* Spin state of ferric iron in MgSiO3 perovskite and its effect on elastic properties. *Earth Planet. Sci. Lett.* **289**, 68–75 (2010).

8. Hsu, H., Blaha, P., Cococcioni, M. & Wentzcovitch, R. M. Spin-state crossover and hyperfine interactions of ferric iron in MgSiO3 perovskite. *Phys. Rev. Lett.* **106**, 1–4 (2011).

9. Lin, J. F. *et al.* Electronic spin states of ferric and ferrous iron in the lower-mantle silicate perovskite. *Am. Mineral.* **97**, 592–597 (2012).

10. Sinmyo, R., McCammon, C. & Dubrovinsky, L. The spin state of Fe3+ in lower mantle bridgmanite. *Am. Mineral.* **102**, 1263–1269 (2017).

11. Liu, J. *et al.* Valence and spin states of iron are invisible in Earth’s lower mantle. *Nat. Commun.* **9**, (2018).

12. Mashino, I. *et al.* Chemistry and mineralogy of earth’s mantle. the spin state of iron in Fe3+-bearing Mg-perovskite and its crystal chemistry at high pressure. *Am. Mineral.* **99**, 1555–1561 (2014).

13. Catalli, K. *et al.* Effects of the Fe3+ spin transition on the properties of aluminous perovskite-New insights for lower-mantle seismic heterogeneities. *Earth Planet. Sci. Lett.* **310**, 293–302 (2011).

14. Fujino, K. *et al.* Spin transition of ferric iron in Al-bearing Mg-perovskite up to 200GPa and its implication for the lower mantle. *Earth Planet. Sci. Lett.* **317**–**318**, 407–412 (2012).

15. Potapkin, V. *et al.* Effect of iron oxidation state on the electrical conductivity of the Earth’s lower mantle. *Nat. Commun.* **4**, 4–9 (2013).

16. Glazyrin, K. *et al.* Magnesium silicate perovskite and effect of iron oxidation state on its bulk sound velocity at the conditions of the lower mantle. *Earth Planet. Sci. Lett.* **393**, 182–186 (2014).

17. Lin, J. F. *et al.* High-spin Fe2+ and Fe3+ in single-crystal aluminous bridgmanite in the lower mantle. *Geophys. Res. Lett.* **43**, 6952–6959 (2016).

18. Hsu, H., Yu, Y. G. & Wentzcovitch, R. M. Spin crossover of iron in aluminous MgSiO3 perovskite and post-perovskite. *Earth Planet. Sci. Lett.* **359**–**360**, 34–39 (2012).

19. Mohn, C. E. & Trønnes, R. G. Iron spin state and site distribution in FeAlO 3 -bearing bridgmanite. *Earth Planet. Sci. Lett.* **440**, 178–186 (2016).

20. Shukla, G., Cococcioni, M. & Wentzcovitch, R. M. Thermoelasticity of Fe3+- and Al-bearing bridgmanite: Effects of iron spin crossover. *Geophys. Res. Lett.* **43**, 5661–5670 (2016).

21. Caracas, R. Elasticity of AlFeO3 and FeAlO3 perovskite and post-perovskite from first-principles calculations. *Geophys. Res. Lett.* **37**, 3–7 (2010).

22. Ballaran, T. B. *et al.* Effect of chemistry on the compressibility of silicate perovskite in the lower mantle. *Earth Planet. Sci. Lett.* **333**–**334**, 181–190 (2012).

23. Ismailova, L. *et al.* Stability of Fe,Al-bearing bridgmanite in the lower mantle and synthesis of pure Fe-bridgmanite. *Sci. Adv.* **2**, (2016).

24. Mao, Z. *et al.* Equation of state and hyperfine parameters of high-spin bridgmanite in the Earth’s lower mantle by synchrotron X-ray diffraction and Mössbauer spectroscopy. *Am. Mineral.* **102**, 357–368 (2017).

25. Kupenko, I. *et al.* Oxidation state of the lower mantle: In situ observations of the iron electronic configuration in bridgmanite at extreme conditions. *Earth Planet. Sci. Lett.* **423**, 78–86 (2015).

26. Zhu, F. *et al.* Synthesis, Elasticity, and Spin State of an Intermediate MgSiO 3 ‐FeAlO 3 Bridgmanite: Implications for Iron in Earth’s Lower Mantle. *J. Geophys. Res. Solid Earth* **125**, (2020).

27. Okuda, Y. *et al.* The effect of iron and aluminum incorporation on lattice thermal conductivity of bridgmanite at the Earth’s lower mantle. *Earth Planet. Sci. Lett.* **474**, 25–31 (2017).

28. Okuda, Y. *et al.* Effect of spin transition of iron on the thermal conductivity of (Fe, Al)-bearing bridgmanite. *Earth Planet. Sci. Lett.* **520**, 188–198 (2019).

29. Nishio-Hamane, D., Seto, Y., Fujino, K. & Nagai, T. Effect of FeAlO3 incorporation into MgSiO3 on the bulk modulus of perovskite. *Phys. Earth Planet. Inter.* **166**, 219–225 (2008).

30. Okuda, Y., Ohta, K., Sinmyo, R., Hirose, K. & Ohishi, Y. Anomalous compressibility in (Fe,Al)-bearing bridgmanite: implications for the spin state of iron. *Phys. Chem. Miner.* **47**, 40 (2020).

31. Shim, S. H. *et al.* Stability of ferrous-iron-rich bridgmanite under reducing midmantle conditions. *Proc. Natl. Acad. Sci. U. S. A.* **114**, 6468–6473 (2017).

32. Dorfman, S. M. *et al.* Effects of composition and pressure on electronic states of iron in bridgmanite. *Am. Mineral.* **105**, 1030–1039 (2020).

33. Frost, D. J. *et al.* Experimental evidence for the existence of iron-rich metal in the Earth’s lower mantle. *Nature* **428**, 409–412 (2004).

34. Zhang, F. & Oganov, A. R. Valence state and spin transitions of iron in Earth’s mantle silicates. *Earth Planet. Sci. Lett.* **249**, 436–443 (2006).

35. Tsuchiya, T. & Wang, X. Ab initio investigation on the high-temperature thermodynamic properties of Fe3+-bearing MgSiO3 perovskite. *J. Geophys. Res. Solid Earth* **118**, 83–91 (2013).
